# Complete mitogenome of *Anopheles sinensis* and mitochondrial insertion segments in the nuclear genomes of 19 mosquito species

**DOI:** 10.1371/journal.pone.0204667

**Published:** 2018-09-27

**Authors:** Yi-Ran Ding, Bo Li, Yu-Juan Zhang, Qi-Meng Mao, Bin Chen

**Affiliations:** 1 Chongqing Key Laboratory of Vector Insects, Chongqing Normal University, Chongqing, P.R. China; 2 Institute of Entomology and Molecular Biology, Chongqing Normal University, Chongqing, P.R. China; Chuo University, JAPAN

## Abstract

*Anopheles sinensis* is a major malarial vector in China and Southeast Asia. The mitochondria is involved in many important biological functions. Nuclear mitochondrial DNA segments (NUMTs) are common in eukaryotic organisms, but their characteristics are poorly understood. We sequenced and analyzed the complete mitochondrial (mt) genome of *An*. *sinensis*. The mt genome is 15,418 bp long and contains 13 protein-coding genes (PCGs), two rRNAs, 22 tRNAs and a large non-coding region. Its gene arrangement is similar to previously published mosquito mt genomes. We identified and analyzed the NUMTs of 19 mosquito species with both nuclear genomes and mt genome sequences. The number, total length and density of NUMTs are significantly correlated with genome size. About half of NUMTs are short (< 200 bp), but larger genomes can house longer NUMTs. NUMTs may help explain genome size expansion in mosquitoes. The expansion due to mitochondrial insertion segments is variable in different insect groups. PCGs are transferred to nuclear genomes at a higher frequency in mosquitoes, but NUMT origination is more different than in mammals. Larger-sized nuclear genomes have longer mt genome sequences in both mosquitoes and mammals. The study provides a foundation for the functional research of mitochondrial genes in *An*. *sinensis* and helps us understand the characteristics and origin of NUMTs and the potential contribution to genome expansion.

## Introduction

Mitochondria are eukaryotic cell organelles that are mainly involved in oxidative phosphorylation [1–2]. The conservation, easy alignment, maternal inheritance, and straightforward gene orthology of mitochondrial (mt) genomes have made the mt genome important for studies of phylogeny and evolution [3–6]. Mt genomes are sometimes associated with insecticide resistance. Several transcripts encoding enzymes such as NADH dehydrogenase and ATP synthase, which are involved in the production of energy within the respiratory chain, were over-expressed in *Aedes aegypti* larvae exposed to insecticides [7]. *Anopheles sinensis* is a major malarial vector in Asia [8], and its mt genome was partially sequenced (15,076 bp) and annotated using collections from Shandong, China [9]. *An*. *sinensis* is being used as a model species to study the molecular mechanism of insecticide resistance [10–11]. To elucidate the function of the mt genome in insecticide resistance, the mt genome need to be completely sequenced and annotated with a laboratory strain originally collected from Jiangsu, China.

Nuclear mitochondrial DNA segments (NUMT) (mitochondrial DNA in the nuclear genome) exist widely in eukaryotes [12]. NUMTs probably arise from nonhomologous recombination of nuclear DNA with mt genome segments from damaged mitochondria [13,14]. The NUMTs found in the nuclear genome are highly similar to mtDNA sequences, so they are easily amplified using universal primers of mtDNA target sequence [15–17]. The existence of NUMTs does not only increases the difficulty of obtaining mtDNA target sequences, but also leads to incorrect conclusions in molecular identification [16–19]. An possible example is the molecular identification of *Dendrocygna arcuate*, which are completely different based on *COI*-NUMTs and *COI*-mtDNA, respectively [20]. At the same time, the transferred mtDNA sequences are thought to be molecular fossils in the nuclear genome and thus identification of NUMTs can help in phylogeny and evolution research [21]. Therefore, NUMTs can be useful molecular markers for the phylogenetic studies of *Homo sapiens* [22] and *Arabidopsis thaliana* [23].

Therefore, the genome-wide identification of NUMTs is very important for the molecular identification and phylogenetic study. NUMTs have been identified in several insect species, including *Atta cephalotes* [24], *Podisma pedestris* [25] and *Sitobion miscanthi* [26]. There is no NUMT in the *An*. *gambiae* genome [21], whereas NUMTs exist in *Culex quinquefasciatus* and *Aedes aegypti* genomes with the NUMT number and total length varying between the two species [27]. The genomes of *An*. *gambiae* [28], *Cx*. *quinquefasciatus* [29], *Aedes aegypti* [30] and *Ae*. *albopictus* [31] have been sequenced and annotated. Recently 16 *Anopheles* genomes have been reported [32]. The nuclear genome of *An*. *sinensis* has been sequenced at the Chongqing Normal University, China. We asked if the number, total length and density of NUMTs are correlated with genome size. Does the mt genome contribute to nuclear genome expansion? Which kinds of genes are most likely to be transferred to the nuclear genome? Sixteen mosquito species, with known phylogeny and known nuclear genome and mt genome sequences, provided us with an opportunity to answer these questions.

We sequenced the complete mt genome of *An*. *sinensis* and analyzed its characteristics, including gene organization, base composition, codon usage, and tRNA secondary structure. We also studied the NUMTs of 19 mosquito species for which both mt genome and nuclear genome sequences are available. The NUMT characteristics, including NUMT number, position, and density in each nuclear genome were determined. This study is significant for the annotation of the complete mt genome of *An*. *sinensis* and for understanding NUMT status and characteristics in mosquitoes.

## Materials and methods

### Sequencing and analysis of *An*. *sinensis* mt genome

The *An*. *sinensis* sample for mt genome sequencing was from an *An*. *sinensis* pyrethroid-susceptible laboratory colony. This colony was established from individuals collected in Wuxi, Jiangsu Province, China and cultured at Chongqing Normal University, China. Mitochondrial genomic DNA was isolated from a single adult female using the sodium dodecyl sulfate (SDS)/proteinase K digestion method [33].

Mitochondrial DNA fragments, ranging in size from 822 bp to 1371 bp, were amplified by PCR using a primer set specific for mosquitoes [34]. A 25-μl PCR reaction was prepared with Takara Ex Taq polymerase (Takara, Japan) and 1.5 μl of 25 mM MgCl_2_, 2.5 μl of 10×PCR Buffer (Mg^2+^ free), 3 μl of a dNTP mixture (2.5 mM each), 1 μl of each 10 mM primer [34], 0.2 μl of 5 U/μl Taq polymerase and 1 μl of the mt genome DNA template. PCR thermal cycling included a 5 min initial denaturation at 94°C for 5 min, followed by 35 cycles of denaturation at 94°C for 1 min, annealing at 48°C–55°C for 45 s, and elongation at 68°C for 1 min, followed by a final elongation for 10 min at 72°C. The PCR products were electrophoresed on a 1.0% agarose gel and then purified using a QIAquick Gel Extraction Kit (QIAGEN, Hilden, Germany). The purified PCR products were directly sequenced using the primer sets except for the control region. The purified products were loaded into pMD-19T vectors, cloned, and then sequenced. All fragments were sequenced in both directions.

The obtained sequences were edited using DANMAN (http://www.lynnon.com/) and were identified in reference to annotated mosquito mt genome sequences through alignment using Clustal X [35]. The sequences of PCGs were translated into amino acids using MEGA version 6.0 [36]. Almost all tRNAs were also recognized by the online tRNAscan-SE Search Server v.1.21 [37], and the tRNAs (*tRNA*^*Arg*^, *tRNA*^*Ser*(AGN)^) that could not be found by tRNAscan-SE were confirmed by sequence homology comparison. The CR was examined for repeats and special structures with the aid of the Tandem Repeats Finder (http://tandem.bu.edu/trf/trf.html) [38]. The nucleotide composition was calculated using DNA Star (http://www.dnastar.com/). Codon usage bias was calculated using MEGA version 6.0 [36]. Strand asymmetry was evaluated by AT Skew and GC Skew using the formulae AT skew = [A%−T%]/ [A%+T%] and GC skew = [G%−C%]/ [G%+C%], respectively [39].

### Nuclear mitochondrial DNA insertion analysis

Eighteen mosquito species’ nuclear genome sequences in FASTA format were downloaded from VectorBase (https://www.vectorbase.org/). The genome sequence of *An*. *sinensis* was obtained from Chongqing Normal University (unpublished data) (S1 Table). The *An*. *sinensis* genome has an assembly scaffold size of 194.49 Mb, with gene area coverage 98.90%. Of the 19 mosquito species with genomes analyzed for NUMTs, three species belonged to the Culicinae with two species in the genus *Aedes* and one in the genus *Culex*. The remaining 16 species were all in the genus *Anopheles* and belonged to the Anophelinae. The 18 previously determined mt genome sequences were downloaded from NCBI, and the mt genome sequence of *An*. *sinensis* was obtained in the present study (S1 Table).

The NUMTs were identified using mt genome sequences to search against the nuclear genome sequence using BLASTN for each species. The significance threshold for the BLASTN search was set to E<10–4, with the window slide of 30 bp [40]. The number, total length and density (NUMTs per Mb of nuclear genome sequence) were calculated with Excel 2010 and used to measure the basic characteristics of NUMTs’ occurrence in the genome. Excel 2010 was also used to count the number and different lengths of the NUMTs. The statistical analyses were carried out using TIBCO Statistica (https://www.tibco.com/products/tibco-statistica). An R package was written to calculate the location and frequency of different areas of the mt genome sequences that had been transferred to the nuclear genome.

## Results and discussion

### *An*. *sinensis* mt genome and its organization

We determined the complete mt genome sequence of *An*. *sinensis*. It is a typical circular and double-stranded molecule of 15,418 bp (GenBank accession MF322628). The obtained mt genome sequence was 342 bp longer than an earlier report of the *An*. *sinensis* mt genome [9], and the difference was mainly due to earlier incomplete sequencing of the mt genome control region (CR). Other genes lengths in our assembly of *An*. *sinensis* mtgenome are same as previous assembly sequence (Genbank accession NC028016) expect for control region. The two mt genome sequences have a 98.6% similarity, and a total of 151 single nucleotide polymorphisms (SNPs) were identified between them, except for CR.

**Fig 1:**
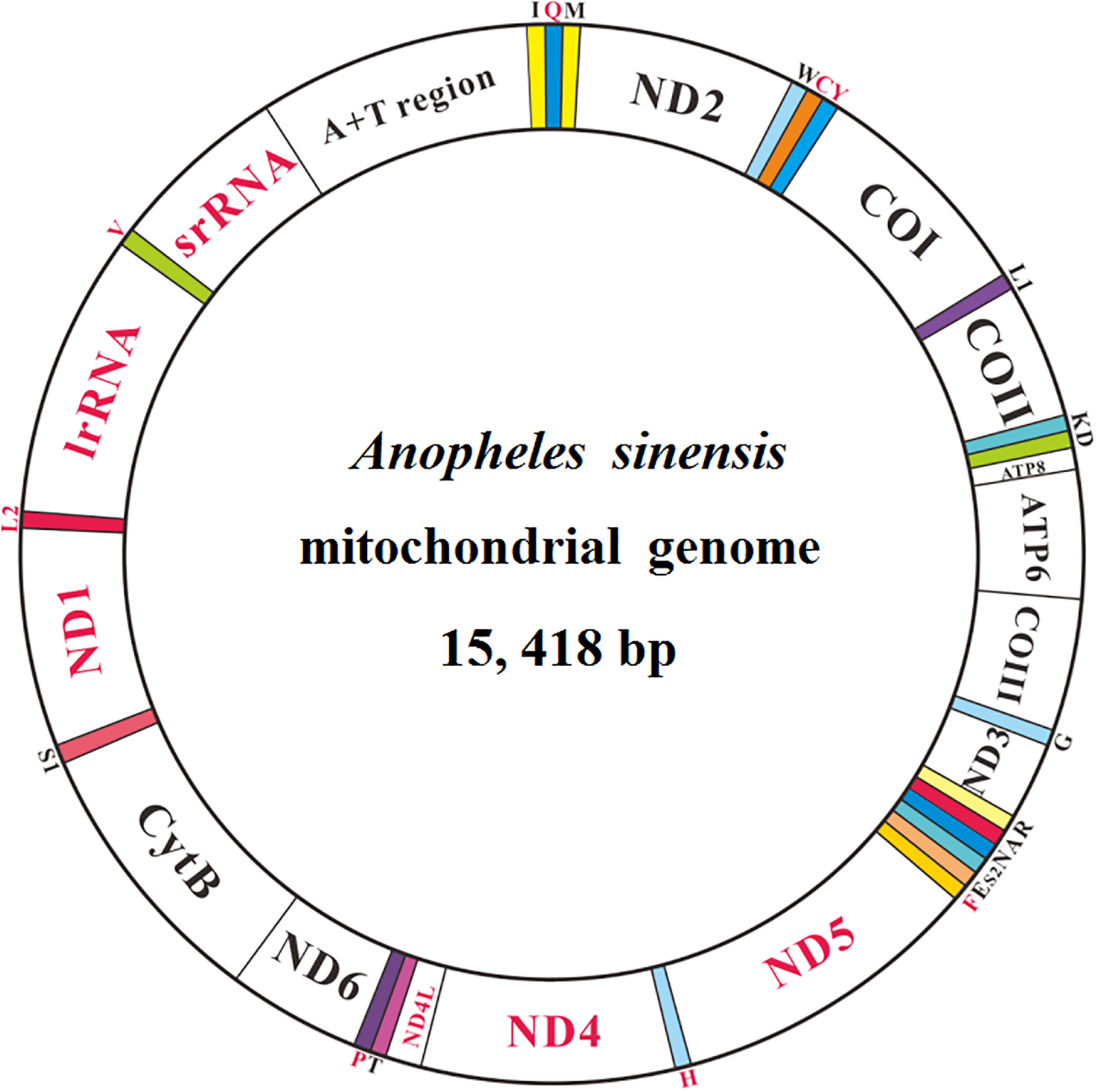
Structure of the *Anopheles sinensis* mt genome. The color-filled blocks indicate tRNAs, and the unfilled white blocks denote protein-coding genes (PCGs), rRNAs and control region (CR). The tRNAs, PCGs, rRNAs and CR located on the majority strand are marked in black, and those on the minority strand are marked in red.

The mt genome sequence of *An*. *sinensis* contains a conserved set of 37 genes, including 13 protein-coding genes (PCGs), two rRNA genes (lrRNA and srRNA), 22 tRNA genes (tRNAs) and a large non-coding region (CR, also known as the AT-rich region) (Fig 1). Twenty-three genes are located on the majority strand (J-strand), while 14 genes reside on the minority strand (N-strand) (Fig 1 and Table 1). The gene arrangement is the same as in previously published mosquito mt genomes, and the unique difference of the arrangement from other dipteran species mt genomes is that the latter have the order trnA-trnR [41]. This arrangement difference might be associated with different adaptations and evolutionary histories of mosquitoes [42].

**Table 1:** Organization of the *Anopheles sinensis* mt genome.

Gene	Position	Strand	Size (bp)	Anticodon	Start codon	Stop codon	No. of intergenic nucleotides
*tRNAIle*	1–68	+	68	GAT			
*tRNAGln*	66–134	-	69	TTG			-3
*tRNAMet*	134–202	+	69	CAT			-1
*ND2*	203–1228	+	1026		ATT	TAA	0
*tRNATrp*	1227–1295	+	69	TCA			-2
*tRNACys*	1295–1358	-	64	GCA			-1
*tRNATyr*	1360–1425	-	66	GTA			1
*COI*	1424–2965	+	1542		TCG	TAA	-2
*tRNALeu* (UUR)	2961–3026	+	66	TAA			-5
*COII*	3028–3712	+	685		ATG	T	1
*tRNALys*	3713–3784	+	72	CTT			0
*tRNAAsp*	3793–3861	+	69	GTC			8
*ATP8*	3862–4023	+	162		ATT	TAA	0
*ATP6*	4017–4697	+	681		ATG	TAA	7
*COIII*	4697–5483	+	787		ATG	T	-1
*tRNAGly*	5484–5550	+	67	TCC			0
*ND3*	5551–5904	+	354		ATA	TAA	0
*tRNAArg*	5903–5966	+	64	TCG			-2
*tRNAAla*	5967–6032	+	66	TGC			0
*tRNAAsn*	6033–6099	+	67	GTT			0
*tRNASer* (AGN)	6102–6168	-	67	GCT			2
*tRNAGlu*	6170–6235	+	66	TTC			1
*tRNAPhe*	6234–6300	-	67	GAA			0
*ND5*	6301–8043	-	1743		GTG	TAA	0
*tRNAHis*	8044–8108	-	65	GTG			0
*ND4*	8106–9450	-	1345		ATG	T	-3
*ND4L*	9444–9743	-	300		ATG	TAA	-7
*tRNAThr*	9750–9814	+	65	TGT			6
*tRNAPro*	9815–9880	-	66	TGG			0
*ND6*	9883–10407	+	527		ATT	TAA	2
*CYTB*	10407–11543	+	1137		ATG	TAA	-1
*tRNASer* (UCN)	11542–11607	+	66	TGA			-2
*ND1*	11628–12572	-	945		ATT	TAA	20
*tRNALeu* (CUN)	12579–12644	-	66	TAG			6
*lrRNA*	12645–13972	-	1328				0
*tRNAVal*	13973–14044	-	72	TAC			0
*srRNA*	14045–14841	-	797				0
CR	14842–15418		577				0

### Characteristics of the *An*. *sinensis* mt genome

The A+T content of *An*. *sinensis* mt genome is 78.34%, and the A+T contents of PCGs, tRNAs, rRNAs and CR are 76.85%, 78.59%, 81.46% and 93.58%, respectively (Table 2). This result is similar to the universal feature presumed from earlier reported mosquito mt genomes [43–51] in that the CR has the highest A+T content, followed by rRNAs. For the 13 PCGs in the *An*. *sinensis* mt genome, the third codon position has a higher A+T content (94.24%), followed by the first codon position (69.46%) and the second codon position (66.84%). The results supports the data from other known mosquito mt genomes [43–51] in that the 3rd codon position has the highest A+T content, followed by the 1st codon position and 2nd codon position.

**Table 2:** Nucleotide composition of the *An*. *sinensis* mt genome.

Mitogenome region	T content %T	C content %C	A content %A	G content %G	A+T content %A+T	AT Skew	GC Skew
Whole genome	38.14	12.50	40.20	9.15	78.34	0.026	-0.155
Protein-coding genes	43.94	11.04	32.90	12.12	76.85	-0.144	0.047
First codon position	37.72	10.93	31.74	19.61	69.46	-0.086	0.284
Second codon position	46.32	19.21	20.52	13.96	66.84	-0.386	-0.158
Third codon position	47.79	2.97	46.45	2.79	94.24	-0.014	-0.033
Protein-coding genes-J	41.52	13.05	34.08	11.36	75.60	-0.098	-0.069
First codon position	34.21	13.18	32.85	19.76	67.06	-0.020	0.200
Second codon position	44.68	21.55	20.99	12.78	65.66	-0.361	-0.255
Third codon position	45.68	4.41	48.39	1.53	94.07	0.029	-0.485
Protein-coding genes-N	47.79	7.84	31.04	13.32	78.83	-0.212	0.259
First codon position	43.30	7.36	29.98	19.36	73.28	-0.182	0.449
Second codon position	48.92	15.48	19.78	15.82	68.70	-0.424	0.011
Third codon position	51.15	0.69	43.37	4.79	94.52	-0.082	0.747
tRNA genes	39.02	9.21	39.57	12.20	78.59	0.007	0.140
tRNA genes-J	37.99	10.53	40.73	10.76	78.72	0.035	0.011
tRNA genes-N	40.53	7.31	37.87	14.29	78.40	-0.034	0.323
rRNA	43.15	6.54	38.31	12.00	81.46	-0.059	0.294
Control region	49.91	3.99	43.67	2.43	93.58	-0.067	-0.243

AT-skew and GC-skew have also been widely used to measure the nucleotide compositional behaviors of mt genomes [42]. The AT skew and GC skew of the *An*. *sinensis* mt genome are 0.026 and -0.155, respectively (Table 2). The AT-skew values are positive, and the GC-skew values are negative for all other mosquito mt genomes [43–51], which indicated overall mt genome preference for the use of A and C. The PCGs of the *An*. *sinensis* mt genome show an overall negative AT-skew (-0.144) and positive GC-skew (0.047). It is a common phenomenon that the PCGs of mosquito mt genomes prefer to use T and G [43–51].

All 13 PCGs in the *An*. *sinensis* mt genome use ATN as the start codon, except for *COI*, which uses the special start codon TCG, and *ND5*, which uses GTG as the start codon. Use of GTG as a start codon has been documented for mtDNA-encoded proteins in various organisms, including *Anopheles* species [52]. All 13 PCGs use the complete stop codon TAA, except for *COII*, *COIII* and *ND4*, which use the incomplete T as a stop codon. There is no other mosquito species with a mt genome that uses TAG as a stop codon [43–51]. The usage bias of amino acids for the 13 PCGs was identified in the *An*. *sinensis* mt genome. Leu has the highest percentage (16.05%), followed by Phe (9.67%), Ser (9.30%) and Ile (9.24%), and Cys has the lowest percentage (1.10%) (S1 Fig). This order is similar to other mosquito mt genomes [43–51]. Leucine has an inferred high usage frequency, and as a hydrophobic amino acid, it can be a component of many transmembrane proteins in the mitochondria.

**Fig 2:**
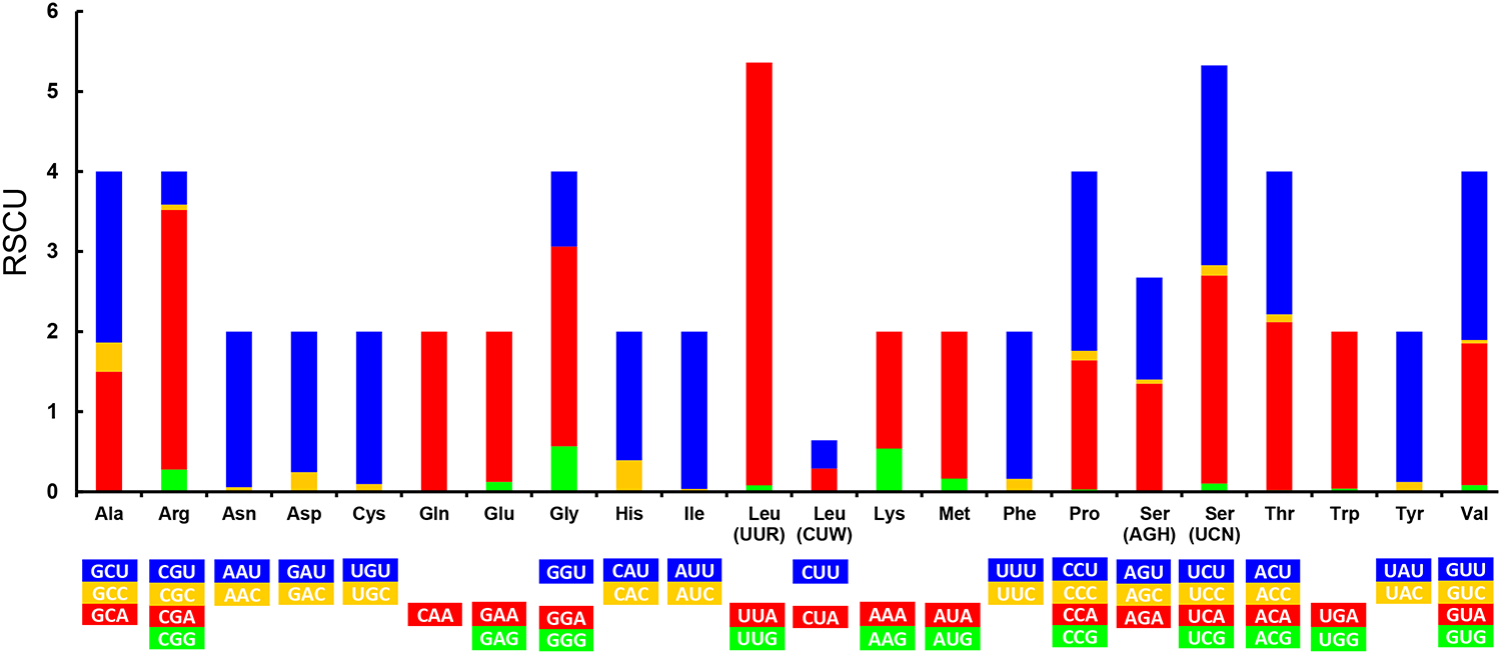
Relative synonymous codon usage (RSCU) in the *An*. *sinensis* mt genome. The RSCU values are represented on the y-axis, while the codon families for each amino acid are denoted on the x-axis.

There are a total of 3733 codons in the *An*. *sinensis* mt genome, excluding termination codons, which is within the codon number range of other insect mt genomes (3585–3746) [53]. For the relative synonymous codon usage (RSCU), UUA is the most used codon (RSCU value 5.28) in the *An*. *sinensis* mt genome, followed by CGA (3.24), UCA (2.60), GGA (2.50), UCU (2.50), CCU (2.24), GCU (2.14), GUU (2.10) and ACA (2.10), and ACG is the least used codon (0.02) (Fig 2). The third codon position has a higher usage frequency of A (46.45%) or U (47.79%) in the *An*. *sinensis* mt genome. This phenomenon is consistent with previously reported mosquito mt genomes [43–51].

All tRNAs of the *An*. *sinensis* mt genome can folded into the typical clover-leaf secondary structure, except for *tRNA*^*Ser*(AGN)^, which has lost the DHU stem (S2 Fig). This is a common feature in metazoan mt genomes [42]. Consistent with other known mosquito mt genomes, all tRNA lengths range from 64 to 72 bp. There are 18 mismatches, all as GU base pairs, found in the 12 tRNAs of the *An*. *sinensis* mt genome (S2 Fig). The large subunit rRNA (*16SrRNA*), 1328 bp long, is located between *ND1* and *tRNA*^*Val*^, and the small subunit rRNA (*12SrRNA*), 797 bp long, is between *tRNA*^*Val*^ and control region, both on the minority strand. The total A+T content of rRNA genes is 81.46%.

The CR plays an important role in the regulation of replication and the transcription of the mt genome [54–55]. The CR region of the *An*. *sinensis* mt genome is 577 bp long and is located between *12SrRNA* and *tRNA*^*Ile*^. The A+T content of this region (93.58%) is higher than other regions of the *An*. *sinensis* mt genome. There is a poly-T stretch of 18 bp to be identified, which may be a recognition site for the initiation of replication in the mt genome [56]. In addition, there are two 46 bp long tandem repeats found in the CR. The tandem repeat structures in CRs are common, but the length and tandem repeat time vary in the other known mosquito mt genomes [6].

### NUMTs and their comparison in mosquitoes

We identified NUMTs of 19 mosquito species with both known mt genomes and nuclear genome sequences. In the subfamily Anophelinae, all 16 species investigated belong to the genus *Anopheles*. Out of the 16, 10 species with genome sizes 141.20–268.44 Mb have no NUMTs, and the remaining 6 species with genome sizes 132.94–217.57 Mb each have 1–3 NUMTs (Table 3). In the subfamily Culicinae, *Cx*. *quinquefasciatus* (genome size 574.57 Mb), *Ae*. *aegypti* (1,342.21 Mb) and *Ae*. *albopictus* (1,868.07 Mb) have 13, 122 and 196 NUMTs, respectively. This suggests that the NUMT number is significantly correlated with genome size (R = 0.9871, *p* = 5.71E-15).

**Table 3:** Number, total length and density of nuclear mitochondrial segments (NUMTs) in 19 mosquito species of nuclear genomes.

Genus/Subgenus/Series	Species	Sizes of mitogenome (kb)/nuclear genome (Mb)	Number/total length (bp) of NUMTs	NUMT density (bp/Mb nuclear genome)
Anophelinae				
*Anopheles*/*Cellia*/Neomyzomyia	*An*. *farauti*^a^	15.412/175.52	2/208	1.19
	*An*. *dirus*	15.404/209.79	0/0	0.00
*Anopheles*/*Cellia*/Neocellia	*An*. *stephensi*	15.387/216.26	0/0	0.00
	*An*. *maculatus*^b^	14.850/141.20	0/0	0.00
*Anopheles*/*Cellia*/Myzomyia	*An*. *minimus*	15.395/195.70	1/43	0.22
	*An*. *culicifacies*	15.364/198.03	0/0	0.00
*Anopheles*/*Cellia*/Pyretophorus	*An*. *christyi*^b^	14.967/169.04	1/309	1.83
	*An*. *epiroticus*	15.379/216.83	0/0	0.00
	*An*. *melas*	15.366/222.01	0/0	0.00
	*An*. *merus*	15.365/244.34	0/0	0.00
	*An*. *coluzzii*	15.441/218.22	0/0	0.00
	*An*. *arabiensis*	15.369/239.13	0/0	0.00
	*An*. *gambiae*	15.363/268.44	0/0	0.00
*Anopheles*/*Anopheles*/Myzorhynchus	*An*. *sinensis*	15.418/194.49	1/216	1.11
*Anopheles*/*Anopheles*/Anopheles	*An*. *atroparvus*	15.458/217.57	3/170	0.78
*Anopheles*/*Nyssorhynchus*/Argyritarsis	*An*. *darlingi*	15.386/132.94	1/122	0.92
Culicinae				
*Culex*/*Culex*/	*Cx*. *quinquefasciatus*	15.587/574.57	13/28,431	51.92
*Aedes*/*Stegomyia*/	*Ae*. *aegypti*	16.655/1,342.21	122/92,934	69.24
	*Ae*. *albopictus*	16.665/1,868.07	196/60,322	32.29

^a^ Lack of partial control region.

^b^ Lack of control region.

The NUMT lengths in the six Anophelinae species range from 43 bp to 309 bp, while lengths in the three Culicinae species range from 37 bp to 15,580 bp. If lengths are divided into three classes (large-size (>2 kb), medium-size (200 bp to 2 kb) and small-size (< 200 bp), it is seen that the large-sized NUMTs only exist in the three Culicinae species, with the largest NUMT (15,580 bp) existing in *Cx*. *quinquefasciatus* (S2 Table). This suggests that larger genomes can house longer NUMTs. If we assemble the NUMTs, the numbers of large-sized, medium-sized and small-sized NUMTs are 12, 157 and 171, respectively. This suggests that half of the NUMTs are small-sized (< 200 bp). Most of the longer NUMTs would be disruptive to shorter NUMTs through nucleotide deletions or insertions in the evolutionary process, which is the probable reason why half of NUMTs are small [26,27].

**Fig 3:**
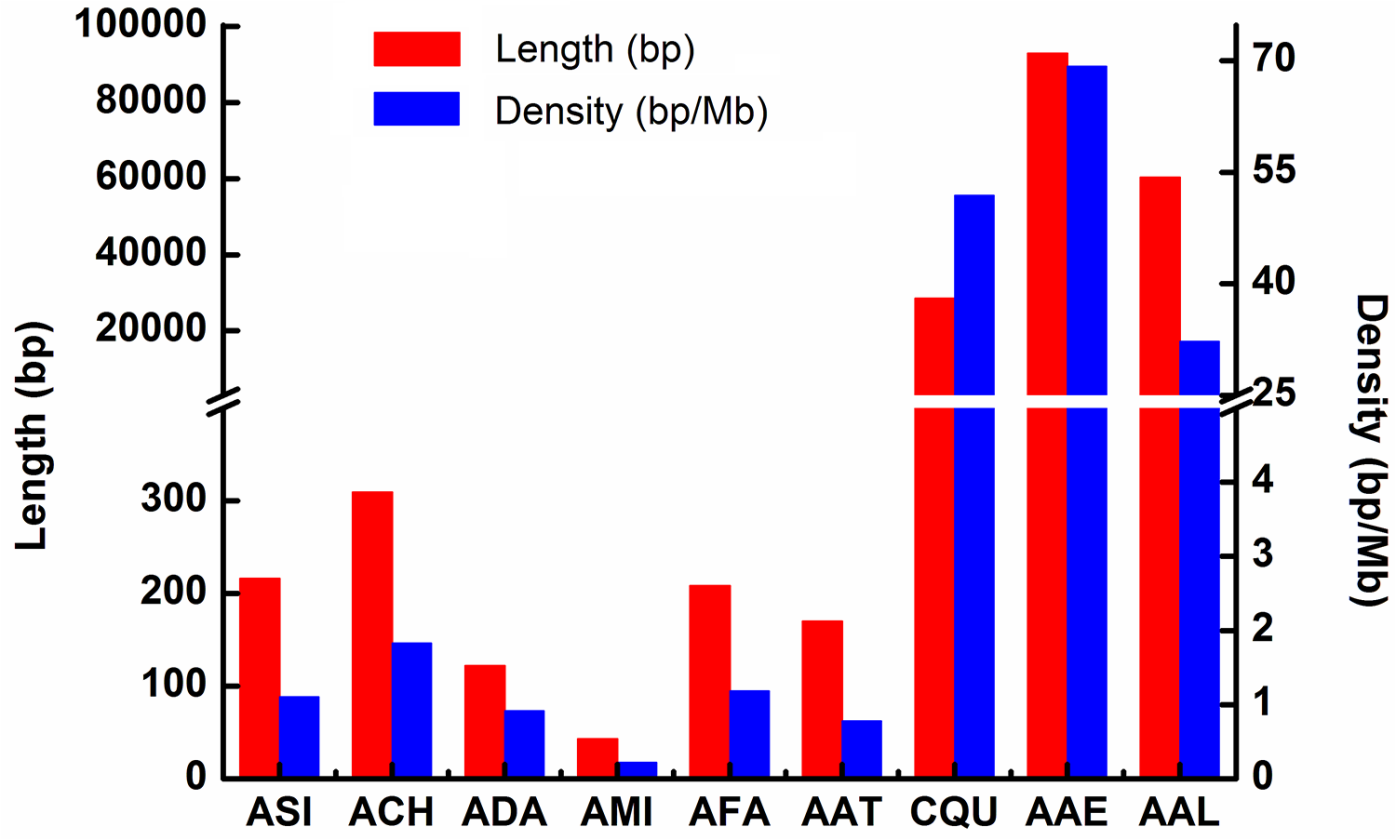
Length (bp) and density (bp/Mb) of nuclear mitochondrial DNA segments (NUMTs) in nine mosquito species. **ASI**: *An*. *sinensis*, **ACH**: *An*. *christyi*, **ADA**: *An*. *darlingi*, **AFA**: *Anopheles farauti*, **AMI**: *An*. *minimus*, **AAT**: *An*. *atroparvus*, **CQU**: *Cx*. *quinquefasciatus*, **AAE**: *Ae*. *aegypti*, **AAL**: *Ae*. *albopictus*.

In the 16 species of Anophelinae, the total length of NUMTs in each species ranges from 0 bp to 309 bp, and the density ranges from 0 bp/Mb to 1.83 bp/Mb of the nuclear genome. The average total length and density are 66.75 bp and 0.38 bp/Mb, respectively (Table 3 and Fig 3). In the three species of Culicinae, the total length of NUMTs in each species range from 28,431 bp to 92,934 bp, and the density range from 32.29 bp/Mb to 69.24 bp/Mb, with the average total length and density being 60,562.33 bp and 51.15 bp/Mb, respectively (Table 3 and Fig 3). The Culicinae species have greater length and density of NUMTs than the Anophelinae species. Statistical analyses showed that the total length of NUMTs is significantly correlated with genome size (R = 0.9104, *p* = 6.31E-08) in all 16 species investigated, which is consistent with the NUMT investigation of 85 species [57]. In addition, the density of NUMTs is significantly correlated with genome size in these mosquitoes (R = 0.7667, *p* = 1.29E-04). The total length and density of NUMTs are also significantly correlated (R = 0.9219, *p* = 2.04E-08). These results suggest that NUMTs could contribute to the expansion of genome size.

In the genetics of other insects, the total length and density of NUMTs in the *Drosophila melanogaster* (genome ~170 Mb), *Tribolium castaneum* (~150 Mb) and *Apis mellifera* (~230 Mb) are 777 bp and 4.57 bp/Mb [58], 8,821 bp and 58.81 bp/Mb [40], and 275,022 bp and 1195.75 bp/Mb [40], respectively. The genome sizes in these three species are comparable with the Anophelinae species; however, the total length and density of NUMTs of the three species are larger than those in the Anophelinae species, especially for *T*. *castaneum* and *A*. *mellifera*. The genome size expansion due to mitochondrial insertion segments is variable in different insect groups. The *A*. *mellifera* nuclear genome recombination rate is much greater than that of *D*. *melanogaster* [59], suggesting that the NUMT total length and density in the nuclear genome is related to the genome recombination rate.

**Fig 4:**
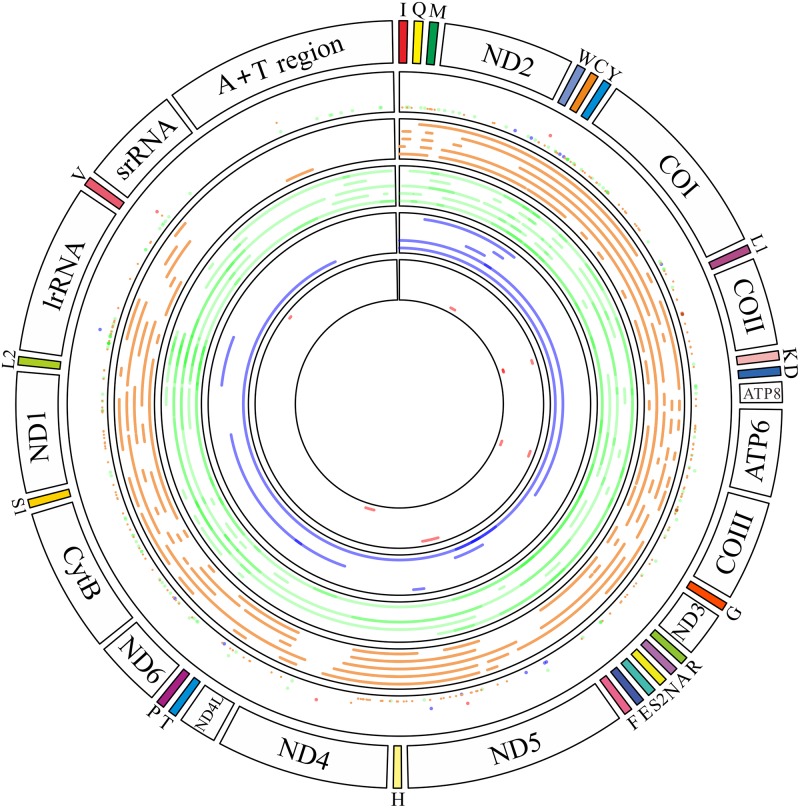
NUMT distribution position in nine mosquito species of mt genomes. The outermost cycle denotes the structure of the reference mt genome, and the second cycle from outside marks the starting positions of the NUMTs for *Ae*. *albopictus* (orange), *Ae*. *aegypti* (green), *Cx*. *quinquefasciatus* (blue) and six *Anopheles* species as a group (red). The four cycles entad show the NUMT regions of the three and six *Anopheles* species as a group, respectively.

The largest number of NUMTs in the genomes of the nine mosquito species originated from the *COI* (39 NUMTs) gene, followed by the *16SrRNA* (31), *CytB* (27) and *ND5* (22) genes. The least number of NUMTs are from the CR (6) (Fig 4). In the six Anophelinae species, NUMTs were derived from only seven mitochondrial genes, including three *COII* in *An*. *atroparvus*, two *COIII* in *An*. *farauti*, one *ND4* in *An*. *sinensis*, one *ND5* in *An*. *christyi*, one *12SrRNA* in *An*. *minimus* and one *tRNA*^*Cys*^-*tRNA*^*Tyr*^ in *An*. *darlingi*. In the three Culicinae species, NUMTs in *Ae*. *aegypti* and *Cx*. *quinquefasciatus* cover the whole mt genome. Similarly, NUMTs in *Ae*. *albopictus* also cover the whole mt genome, with the exception of *ND4*, *12SrRNA* and CR. These data suggest that the PCGs are transferred to the nuclear genome at a higher frequency, and the larger-sized nuclear genomes in mosquitoes have larger-sized mt genome sequences.

The largest numbers of NUMTs were derived from the *16SrRNA* gene in three mammals, *Sus scrofa* (6), *Pan troglodytes* (65) and *Homo sapiens* (53). In *Mus musculus*, the largest number of NUMTs originated from CR (11), followed by *ND2* (7) and *ND4* (6), but no NUMT was found from *tRNA*^*Val*^ [60]. This suggests that the NUMT origination may be different in mosquitoes and mammals. The genome sizes of *M*. *musculus*, *S*. *scrofa*, *P*. *troglodytes* and *H*. *sapiens* are 2.7 G, 2.3 G, 2.9 G and 2.9 G, and the mt genome coverage rates of NUMT are 84.7%, 32.6%, 100% and 100%, respectively [60]. The larger-sized nuclear genomes in mammals appear to have a wider range of mt genome sequence coverage rates.

## Conclusion

We studied mitochondrial genes in *An*. *sinensis* through analysis of the complete mt genome sequence. NUMT analysis of nineteen mosquito species led to the conclusion that the number, total length and density of NUMTs are significantly correlated with genome size. NUMTs are an important cause of nuclear genome size expansion in mosquitoes. The genome size expansion due to mitochondrial insertion segments is variable in different insect groups. PCGs are transferred to the nuclear genome at a higher frequency in mosquitoes, but the NUMT origination is quite different from mammals. Larger-sized nuclear genomes, in both mosquitoes and mammals, have a wider range of transferred mt genome sequences.

## Supporting information

S1 Fig The percentage of each amino acid in the *An*. *sinensis* mt genome. N-strand: majority strand; J-strand: minority strand.
(TIF)

S2 Fig Inferred secondary structures of tRNAs in the *An*. *sinensis* mt genome. The tRNAs are labeled with their corresponding amino acids.
(TIF)

S1 Table Mt genome and nuclear genome sequence information of 19 mosquito species. (DOC)

S2 Table Positions and lengths of NUMTs in the nuclear genomes of nine species. (DOC)
