# Supplementary figures and images for: Complete mitogenome of Anopheles sinensis and mitochondrial insertion segments in the nuclear genomes of 19 mosquito species
Source: PLoS One. 2018 Sep 27;13(9):e0204667. doi: 10.1371/journal.pone.0204667 (PMC6160108; doi:10.1371/journal.pone.0204667)

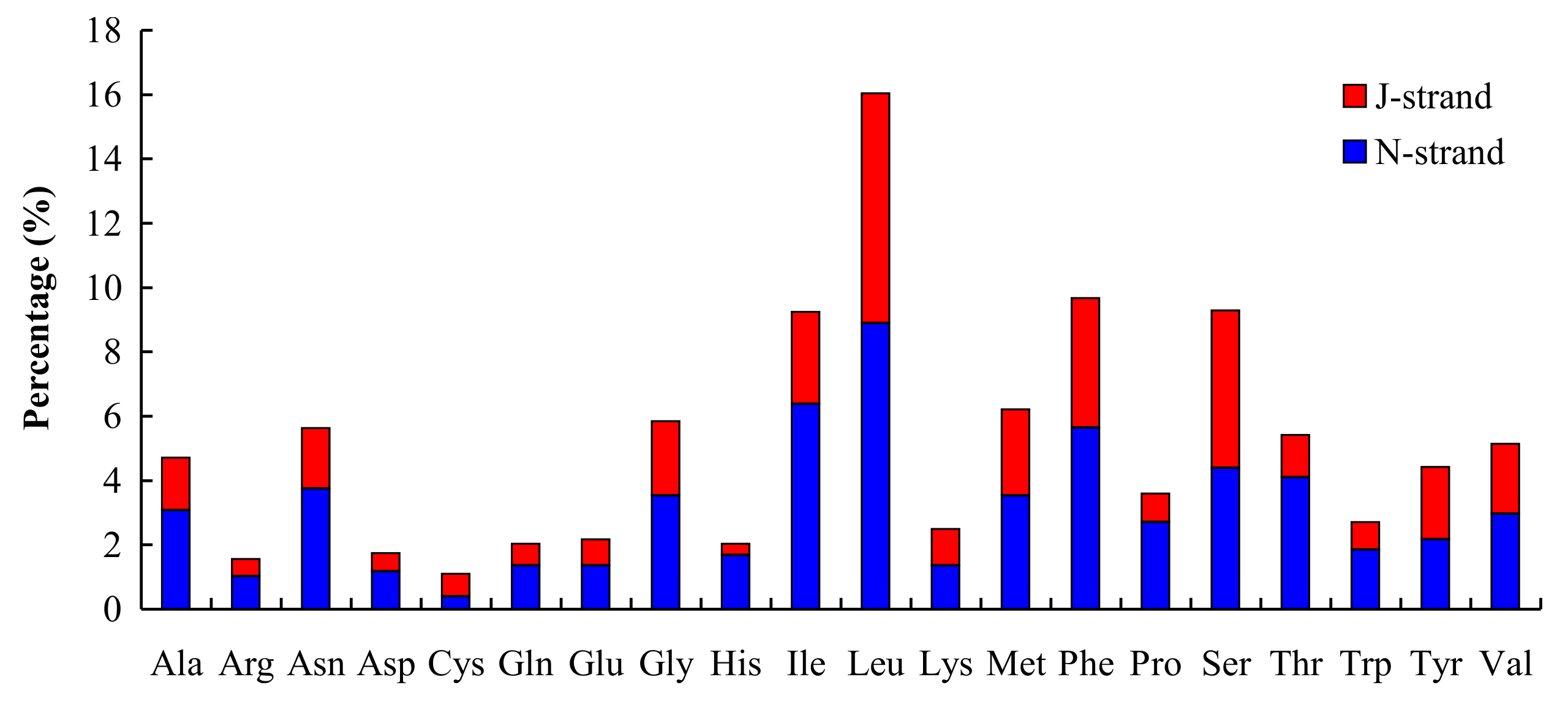

Supplement: S1 Fig — N-strand: majority strand; J-strand: minority strand. (TIF) [file pone.0204667.s001.tif]

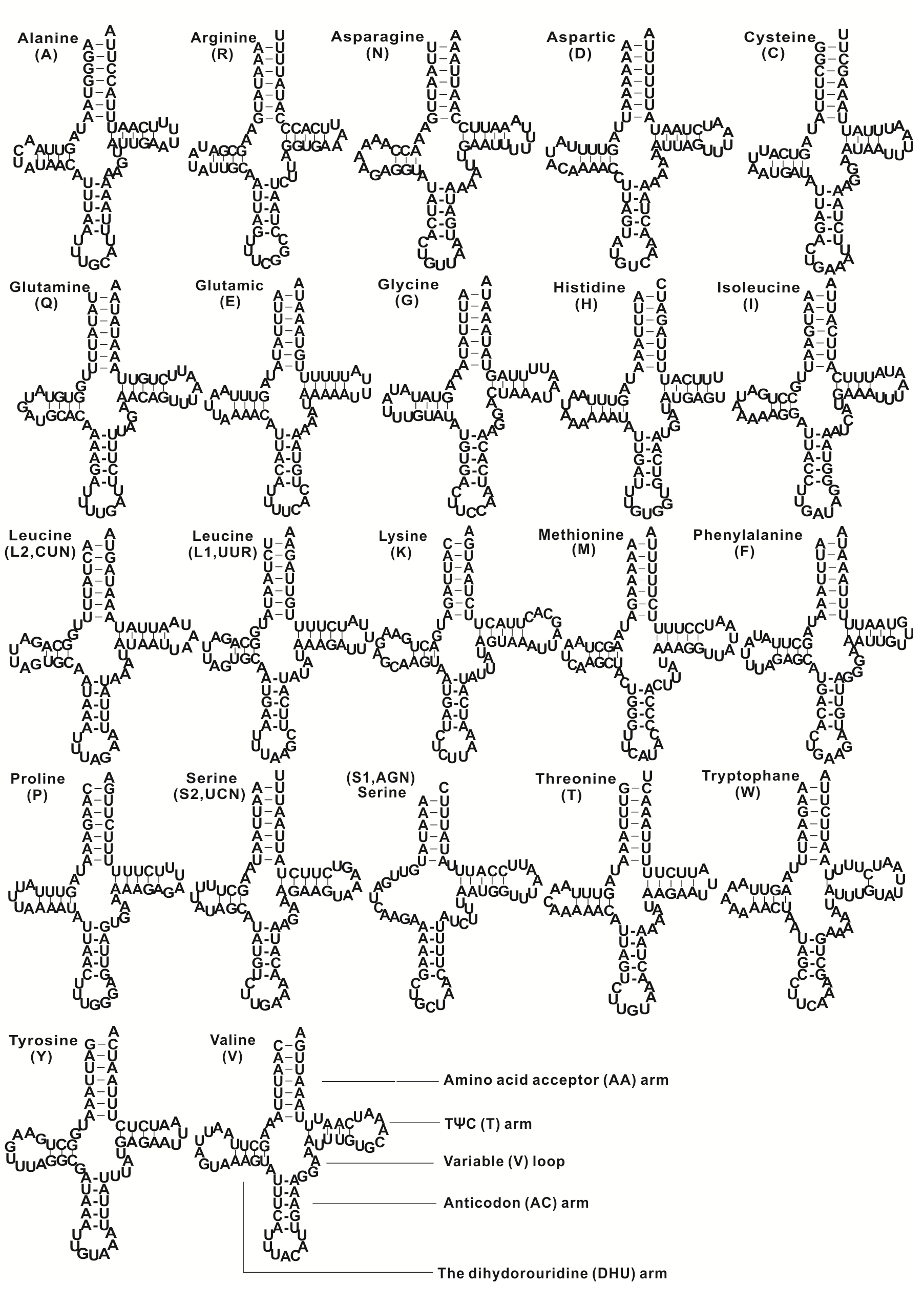

Supplement: S2 Fig — The tRNAs are labeled with their corresponding amino acids. (TIF) [file pone.0204667.s002.tif]
