# Supplementary material for: Complete mitogenome of Anopheles sinensis and mitochondrial insertion segments in the nuclear genomes of 19 mosquito species
Source: PLoS One. 2018 Sep 27;13(9):e0204667. doi: 10.1371/journal.pone.0204667 (PMC6160108; doi:10.1371/journal.pone.0204667)
Supplement: S1 Table — (DOC) [file pone.0204667.s003.doc]

**Table S1 Mtgenome and nuclear genome sequence information of 19 mosquito species.**

| **Genus/Subgenus/Series** | **Species** | **Mtgenome source ^a^** | | | **Nuclear genome source ^b^** | | |
| --- | --- | --- | --- | --- | --- | --- | --- |
|  |  | **Accession Number** | **Size (bp)** | **References** | **Version** | **Size (Mb)** | **References** |
| **Anophelinae** |  |  |  |  |  |  |  |
| *Anopheles*/*Cellia*/Neomyzomyia | *An. farauti* | JX219741 | 15412 | Logue, *et al.*^1^ | AfarF1 | 175.52 | Neafsey *et al*.^6^ |
|  | *An. dirus* | JX219731 | 15404 | Logue, *et al.*^1^ | AdirW1 | 209.79 | Neafsey *et al*.^6^ |
| *Anopheles/Cellia*/Neocellia | *An. stephensi* | NC028223 | 15387 | Direct Submission | AsteI2 | 216.26 | Neafsey *et al*.^6^ |
|  | *An. maculatus* | NC028218 | 14850 | Direct Submission | AmacM1 | 141.20 | Neafsey *et al*.^6^ |
| *Anopheles/Cellia*/Myzomyia | *An. minimus* | KT895423 | 15395 | Hua, *et al.*^2^ | AminM1 | 195.70 | Neafsey *et al*.^6^ |
|  | *An. culicifacies* | NC028216 | 15364 | Direct Submission | AculA1 | 198.03 | Neafsey *et al*.^6^ |
| *Anopheles/Cellia*/Pyretophorus | *An. christyi* | NC028214 | 14967 | Direct Submission | AchrA1 | 169.04 | Neafsey *et al*.^6^ |
|  | *An. epiroticus* | NC028217 | 15379 | Direct Submission | AepiE1 | 216.83 | Neafsey *et al*.^6^ |
|  | *An. melas* | NC028219 | 15366 | Direct Submission | AmelC1 | 222.01 | Neafsey *et al*.^6^ |
|  | *An. merus* | NC028220 | 15365 | Direct Submission | AmerM1 | 244.34 | Neafsey *et al*.^6^ |
|  | *An. coluzzii* | NC028215 | 15441 | Direct Submission | AcolM1 | 218.22 | Direct Submission |
|  | *An. arabiensis* | NC028212 | 15369 | Direct Submission | AaraD1 | 239.13 | Neafsey *et al*.^6^ |
|  | *An. gambiae* | NC002084 | 15363 | Beard, *et al.*^3^ | AgamP4 | 268.44 | Holt *et al*.^7^ |
| *Anopheles/Anopheles*/Myzorhynchus | *An. sinensis* | MF322628 | 15418 | This study | unpublished | 194.49 | This study |
| *Anopheles/Anopheles*/Anopheles | *An. atroparvus* | NC028213 | 15458 | Direct Submission | AatrE1 | 217.57 | Neafsey *et al*.^6^ |
| *Anopheles/Nyssorhynchus*/Argyritarsis | *An. darling* | GQ918272 | 15386 | Moreno, *et al.*^4^ | AdarC3 | 132.94 | Neafsey *et al*.^6^ |
| **Culicinae** |  |  |  |  |  |  |  |
| *Culex/Culex*/ | *Cx. quinquefasciatus* | NC014574 | 15587 | Behura, *et al*.^5^ | CpipJ2 | 574.57 | Arensburger *et al*.^8^ |
| *Aedes/Stegomyia/* | *Ae. albopictus* | NC006817 | 16665 | Direct Submission | AaloF1 | 1 868.07 | Chen *et al*.^9^ |
|  | *Ae. aegypti* | NC010241 | 16655 | Behura, *et al.*^5^ | AaegL3 | 1 342.21 | Nene *et al*.^10^ |

^a^ All data of mtgenomes were downloaded from NCBI expect for *An. sinensis* mtgenome, which was reported for the first time in this study.

^b^ All data of genomes came from VectorBase (https://www.vectorbase.org/downloads) and assembled into Scaffolds expect for *An. sinensis* genome, which was sequenced and assembled in the Chongqing Normal University and will be published recently.

**References**

[1] Logue, K., Chan, E. R., Phipps, T., Small, S. T., Reimer, L., & Henryhalldin, C., et al. (2013). Mitochondrial genome sequences reveal deep divergences among *Anopheles punctulatus* sibling species in papua new guinea. *Malaria Journal,* 12(1), 64.

[2] Hua, Y. Q., Ding, Y. R., Yan, Z. T., Si, F. L., Luo, Q. C., & Chen, B. (2016). The complete mitochondrial genome of *Anopheles minimus* (Diptera: Culicidae) and the phylogenetics of known *Anopheles* mitogenomes. *Insect Science*, 23(3), 353-365.

[3] Beard, C. B., Hamm, D. M., & Collins, F. H. (1993). The mitochondrial genome of the mosquito *Anopheles gambiae*: DNA sequence, genome organization, and comparisons with mitochondrial sequences of other insects. *Insect Molecular Biology,* 2(2), 103-124.

[4] Moreno, M., Marinotti, O., Krzywinski, J., Tadei, W. P., James, A. A., & Achee, N. L., et al. (2010). Complete mtDNA genomes of *Anopheles darlingi* and an approach to anopheline divergence time. *Malaria Journal,* 9(1), 127.

[5] Behura, S. K., Lobo, N. F., Haas, B., Debruyn, B., Lovin, D. D., & Shumway, M. F., et al. (2011). Complete sequences of mitochondria genomes of *Aedes aegypti* and *Culex quinquefasciatus* and comparative analysis of mitochondrial dna fragments inserted in the nuclear genomes. *Insect Biochemistry & Molecular Biology*, 41(10), 770.

[6] Neafsey, D.E., Waterhouse, R.M., Abai, M.R., Aganezov, S.S., Alekseyev, M.A., Allen, J.E., Amon, J., Arensburger, P. and Artemov, G. (2015) Highly evolvable malaria vectors: The genomes of 16 *Anopheles* mosquitoes. Science, 347, 1258522.

[7] Holt, R.A., Subramanian, G.M., Halpern, A., Sutton, G.G., Charlab, R., Nusskern, D.R., Wincker, P., Clark, A.G., Ribeiro, J.C. and Wides, R. (2002) The genome sequence of the malaria mosquito *Anopheles gambiae*. Science, 298, 129-149.

[8] Arensburger, P., Megy, K., Waterhouse, R.M., Abrudan, J., Amedeo, P., Antelo, B., Bartholomay, L., Bidwell, S., Caler, E. and Camara, F. (2010) Sequencing of *Culex quinquefasciatus* establishes a platform for mosquito comparative genomics. Science, 330, 86-88.

[9] Chen, X.-G., Jiang, X., Gu, J., Xu, M., Wu, Y., Deng, Y., Zhang, C., Bonizzoni, M., Dermauw, W. and Vontas, J. (2015) Genome sequence of the Asian Tiger mosquito, *Aedes albopictus*, reveals insights into its biology, genetics, and evolution. Proceedings of the National Academy of Sciences, 112, 5907-5915.

[10] Nene, V., Wortman, J.R., Lawson, D., Haas, B., Kodira, C., Tu, Z.J., Loftus, B., Xi, Z., Megy, K. and Grabherr, M. (2007) Genome sequence of *Aedes aegypti*, a major arbovirus vector. Science, 316, 1718-1723.
