# Supplementary material for: Complete mitogenome of Anopheles sinensis and mitochondrial insertion segments in the nuclear genomes of 19 mosquito species
Source: PLoS One. 2018 Sep 27;13(9):e0204667. doi: 10.1371/journal.pone.0204667 (PMC6160108; doi:10.1371/journal.pone.0204667)
Supplement: S2 Table — (DOC) [file pone.0204667.s004.doc]

**Table S2 Postions and length of NUMTs in nine species of nuclear genomes.**

| **Genome position of NUMTs** | **Position on mtgenome** | **NUMT length (bp)** | **E-value** |
| --- | --- | --- | --- |
| ***Anopheles sinensis* :** | | | |
| scaffold 15:2758761-2758976 | 8977-9192 | 216 | 2.00E-108 |
| ***Anopheles christyi*** ^a^**:** | | | |
| KB694098:363-671 | 7604-7912 | 309 | 1.00E-153 |
| ***Anopheles darlingi* :** | | | |
| scaffold_669:2090-2211 | 1304-1422 | 122 | 9.00E-33 |
| ***Anopheles minimus* :** | | | |
| KB664277:288778-288820 | 14297-14339 | 43 | 2.00E-10 |
| ***Anopheles farauti* :** | | | |
| KI915058:582090-581987 | 5099-5202 | 104 | 2.00E-24 |
| KI915058:582219-582116 | 5099-5202 | 104 | 2.00E-24 |
| ***Anopheles atroparvus* :** | | | |
| KI421900:2256834-2256772 | 3331-3393 | 63 | 9.00E-23 |
| KI421900:2256897-2256835 | 3331-3393 | 63 | 9.00E-23 |
| KI421900:2256771-2256728 | 3331-3374 | 44 | 2.00E-12 |
| ***Culex quinquefasciatus* :** | | | |
| supercont3.816:413-15992 | 1-15587 | 15580 | 0 |
| supercont3.816:15999-21743 | 1-5749 | 5745 | 0 |
| supercont3.1381:1-2830 | 9218-12054 | 2830 | 0 |
| supercont3.1381:5317-6056 | 12812-13552 | 740 | 0 |
| supercont3.507:356204-357611 | 363-1768 | 1408 | 0 |
| supercont3.398:274851-275555 | 6588-7366 | 705 | 0 |
| supercont3.181:166063-166529 | 6935-7430 | 467 | 9.00E-148 |
| supercont3.228:5845-6133 | 1065-1385 | 289 | 4.00E-83 |
| supercont3.228:5678-5847 | 1479-1643 | 170 | 3.00E-53 |
| supercont3.429:186086-186241 | 8002-8154 | 156 | 1.00E-58 |
| supercont3.622:228233-228370 | 6927-7064 | 138 | 5.00E-56 |
| supercont3.1139:9551-9652 | 1469-1570 | 102 | 2.00E-43 |
| supercont3.909:17699-17799 | 9983-10083 | 101 | 1.00E-38 |
| ***Aedes aegypti* :** | | | |
| supercont1.363:1110349-1124682 | 1262-15598 | 14334 | 0 |
| supercont1.593:1-12879 | 2899-15779 | 12879 | 0 |
| supercont1.600:574435-582067 | 1-7639 | 7633 | 0 |
| supercont1.600:573138-574434 | 15271-16655 | 1297 | 0 |
| supercont1.765:566937-574709 | 120-7910 | 7773 | 0 |
| supercont1.765:574716-574808 | 5294-5386 | 93 | 1.00E-36 |
| supercont1.1330:113872-118789 | 7448-12361 | 4918 | 0 |
| supercont1.357:533538-537514 | 3181-7159 | 3977 | 0 |
| supercont1.118:1399129-1401697 | 10611-13222 | 2569 | 0 |
| supercont1.118:1403729-1405624 | 14619-16618 | 1896 | 0 |
| supercont1.118:1402642-1403287 | 13975-14620 | 646 | 0 |
| supercont1.118:1406622-1406789 | 1-167 | 168 | 2.00E-73 |
| supercont1.118:118430-118507 | 12727-12804 | 78 | 4.00E-30 |
| supercont1.556:22730-25139 | 10680-13088 | 2410 | 0 |
| supercont1.929:838-2616 | 2039-3818 | 1779 | 0 |
| supercont1.929:6860-8499 | 4992-6630 | 1640 | 0 |
| supercont1.929:2821-4008 | 3813-5000 | 1188 | 0 |
| supercont1.929:5-844 | 559-1398 | 840 | 0 |
| supercont1.368:182607-184207 | 7335-8931 | 1601 | 0 |
| supercont1.36:2226087-2227303 | 13368-14608 | 1217 | 0 |
| supercont1.36:2228051-2228333 | 1871-2153 | 283 | 6.00E-117 |
| supercont1.41:2928545-2929344 | 5975-6781 | 800 | 0 |
| supercont1.194:495376-496582 | 13362-14611 | 1207 | 0 |
| supercont1.194:496663-496885 | 13148-13371 | 223 | 1.00E-87 |
| supercont1.960:244065-244971 | 2470-3373 | 907 | 0 |
| supercont1.90:2645617-2646245 | 38-667 | 629 | 0 |
| supercont1.90:2645439-2645596 | 16470-16628 | 158 | 9.00E-58 |
| supercont1.90:2067369-2067438 | 11280-11349 | 70 | 4.00E-24 |
| supercont1.260:152310-152848 | 13034-13582 | 539 | 0 |
| supercont1.440:877482-877900 | 12833-13251 | 419 | 0 |
| supercont1.440:348372-348469 | 11425-11522 | 98 | 7.00E-34 |
| supercont1.156:2041781-2042186 | 1062-1467 | 406 | 0 |
| supercont1.554:78003-78584 | 372-992 | 582 | 0 |
| supercont1.554:77364-77934 | 12792-13377 | 571 | 2.00E-129 |
| supercont1.554:78590-78885 | 1247-1550 | 296 | 3.00E-77 |
| supercont1.554:73821-74076 | 13031-13290 | 256 | 7.00E-72 |
| supercont1.481:584735-585172 | 15859-16311 | 438 | 8.00E-166 |
| supercont1.481:584527-585172 | 15930-16647 | 646 | 3.00E-145 |
| supercont1.348:814075-814543 | 12228-12719 | 469 | 1.00E-162 |
| supercont1.287:517376-517843 | 12229-12719 | 468 | 5.00E-162 |
| supercont1.287:517843-518388 | 12792-13371 | 546 | 3.00E-121 |
| supercont1.287:515920-516085 | 12994-13154 | 166 | 2.00E-54 |
| supercont1.206:769400-769742 | 2473-2812 | 343 | 1.00E-143 |
| supercont1.763:415176-415557 | 10196-10579 | 382 | 6.00E-142 |
| supercont1.763:414731-414971 | 10577-10817 | 241 | 5.00E-86 |
| supercont1.734:165708-166014 | 9199-9506 | 307 | 3.00E-140 |
| supercont1.454:604405-604713 | 1320-1628 | 309 | 3.00E-140 |
| supercont1.454:604711-604922 | 6260-6474 | 212 | 1.00E-88 |
| supercont1.491:872554-872923 | 10670-11040 | 370 | 3.00E-139 |
| supercont1.11:2777911-2778266 | 10971-11330 | 356 | 2.00E-135 |
| supercont1.2991:3085-3481 | 4553-4946 | 397 | 3.00E-134 |
| supercont1.240:1309519-1310074 | 12792-13371 | 556 | 9.00E-134 |
| supercont1.240:1313936-1314245 | 12994-13290 | 310 | 2.00E-110 |
| supercont1.240:1310074-1310372 | 12399-12719 | 299 | 5.00E-99 |
| supercont1.240:1309213-1309437 | 372-594 | 225 | 7.00E-72 |
| supercont1.607:544646-544981 | 5454-5826 | 336 | 4.00E-132 |
| supercont1.607:672810-672891 | 5100-5181 | 82 | 1.00E-30 |
| supercont1.894:28480-29035 | 12792-13371 | 556 | 2.00E-129 |
| supercont1.894:33697-34015 | 12994-13300 | 319 | 1.00E-106 |
| supercont1.894:28324-28397 | 372-443 | 74 | 1.00E-17 |
| supercont1.822:5757-6044 | 6440-6727 | 288 | 3.00E-127 |
| supercont1.62:2332332-2332884 | 12792-13371 | 553 | 8.00E-122 |
| supercont1.62:2324539-2324846 | 12994-13288 | 278 | 1.00E-106 |
| supercont1.62:2332155-2332332 | 12524-12719 | 178 | 4.00E-49 |
| supercont1.1088:117180-117487 | 1602-1909 | 306 | 2.00E-111 |
| supercont1.23:312739-312991 | 16061-16311 | 253 | 6.00E-104 |
| supercont1.522:623699-623897 | 14531-14729 | 199 | 4.00E-94 |
| supercont1.1225:140331-140540 | 63-275 | 210 | 5.00E-93 |
| supercont1.458:369013-369215 | 1781-1983 | 203 | 5.00E-80 |
| supercont1.551:480830-481053 | 7493-7716 | 224 | 9.00E-77 |
| supercont1.253:496050-496248 | 6273-6473 | 199 | 2.00E-71 |
| supercont1.459:680228-680383 | 4398-4553 | 156 | 8.00E-71 |
| supercont1.98:734527-734738 | 1496-1710 | 212 | 1.00E-68 |
| supercont1.98:752508-752719 | 1496-1710 | 212 | 1.00E-68 |
| supercont1.98:767082-767293 | 1496-1710 | 212 | 1.00E-68 |
| supercont1.98:792849-793060 | 1496-1710 | 212 | 1.00E-68 |
| supercont1.640:625438-625634 | 9843-10041 | 197 | 1.00E-67 |
| supercont1.309:472664-472876 | 10710-10925 | 213 | 4.00E-62 |
| supercont1.405:341187-341356 | 3997-4167 | 170 | 2.00E-60 |
| supercont1.10:3766238-3766389 | 1397-1549 | 152 | 3.00E-57 |
| supercont1.407:81601-81731 | 10286-10420 | 131 | 1.00E-48 |
| supercont1.495:486627-486763 | 1993-2141 | 137 | 2.00E-47 |
| supercont1.739:457521-457656 | 1897-2032 | 136 | 2.00E-46 |
| supercont1.181:1893160-1893294 | 3295-3429 | 135 | 4.00E-43 |
| supercont1.108:1876643-1876737 | 2819-2913 | 95 | 3.00E-39 |
| supercont1.724:459373-459470 | 10533-10630 | 97 | 3.00E-39 |
| supercont1.222:1148365-1148461 | 11104-11200 | 97 | 9.00E-39 |
| supercont1.314:295367-295465 | 2023-2121 | 99 | 4.00E-37 |
| supercont1.109:414681-414780 | 4925-5024 | 100 | 1.00E-36 |
| supercont1.136:1660986-1661075 | 14210-14299 | 90 | 6.00E-35 |
| supercont1.55:2658873-2658958 | 13192-13277 | 86 | 2.00E-34 |
| supercont1.55:742248-742321 | 11215-11288 | 74 | 6.00E-28 |
| supercont1.857:199898-199981 | 10378-10461 | 84 | 2.00E-33 |
| supercont1.433:635952-636043 | 14224-14315 | 92 | 2.00E-33 |
| supercont1.8:3317954-3318053 | 1629-1728 | 100 | 3.00E-32 |
| supercont1.207:981211-981328 | 14289-14406 | 118 | 4.00E-31 |
| supercont1.33:1205041-1205124 | 1152-1236 | 84 | 4.00E-31 |
| supercont1.6:2460253-2460344 | 11441-11532 | 92 | 1.00E-30 |
| supercont1.53:2063259-2063342 | 5018-5101 | 84 | 1.00E-30 |
| supercont1.661:458008-458094 | 3061-3147 | 87 | 1.00E-29 |
| supercont1.63:1107567-1107652 | 11164-11247 | 86 | 1.00E-29 |
| supercont1.2836:2479-2550 | 10743-10814 | 72 | 8.00E-27 |
| supercont1.1:5589008-5589095 | 2803-2890 | 88 | 9.00E-26 |
| supercont1.344:789638-789710 | 5369-5441 | 73 | 9.00E-26 |
| supercont1.569:203453-203522 | 6968-7037 | 70 | 4.00E-24 |
| supercont1.569:203384-203452 | 1633-1701 | 69 | 2.00E-22 |
| supercont1.399:410887-410961 | 4733-4807 | 75 | 4.00E-24 |
| supercont1.1185:143607-143675 | 11280-11348 | 69 | 1.00E-23 |
| supercont1.482:720005-720092 | 185-272 | 88 | 5.00E-23 |
| supercont1.396:148986-149053 | 2034-2101 | 68 | 5.00E-23 |
| supercont1.198:1092954-1093021 | 14180-14247 | 68 | 5.00E-23 |
| supercont1.124:1908660-1908738 | 13150-13228 | 79 | 2.00E-22 |
| supercont1.238:58015-58088 | 505-577 | 74 | 2.00E-21 |
| supercont1.660:36369-36437 | 4606-4674 | 69 | 7.00E-21 |
| supercont1.86:978622-978684 | 162-224 | 63 | 3.00E-20 |
| supercont1.72:2921875-2921948 | 5191-5266 | 74 | 9.00E-20 |
| supercont1.1110:47790-47852 | 16070-16132 | 63 | 3.00E-19 |
| supercont1.889:142929-142990 | 3988-4049 | 62 | 5.00E-17 |
| supercont1.248:1295863-1295925 | 10310-10372 | 63 | 2.00E-16 |
| supercont1.84:1263110-1263147 | 16520-16557 | 38 | 2.00E-08 |
| supercont1.272:778093-778145 | 4012-4065 | 53 | 8.00E-08 |
| supercont1.25:1430922-1430959 | 2038-2075 | 38 | 9.00E-07 |
| ***Aedes albopictus* :** | | | |
| JXUM01S002504:204914-207551 | 620-3245 | 2637 | 0 |
| JXUM01S002504:200588-201622 | 7562-8596 | 1035 | 0 |
| JXUM01S002504:203788-204947 | 5658-6855 | 1160 | 0 |
| JXUM01S002504:207809-207883 | 549-623 | 75 | 1.00E-25 |
| JXUM01S000059:355844-357593 | 11270-13006 | 1750 | 0 |
| JXUM01S023063:1-1689 | 1470-3160 | 1689 | 0 |
| JXUM01S022730:1-1617 | 1615-3267 | 1617 | 0 |
| JXUM01S022730:1619-1768 | 1562-1710 | 150 | 7.00E-61 |
| JXUM01S026382:1-1517 | 1180-2690 | 1517 | 0 |
| JXUM01S005757:65582-66872 | 9296-10584 | 1291 | 0 |
| JXUM01S009535:1-1352 | 3875-5281 | 1352 | 0 |
| JXUM01S004322:35255-36130 | 432-1307 | 876 | 0 |
| JXUM01S003600:2-939 | 7143-8080 | 938 | 0 |
| JXUM01S003600:3040-3350 | 8084-8393 | 311 | 2.00E-130 |
| JXUM01S007548:64063-64908 | 432-1277 | 846 | 0 |
| JXUM01S011970:19722-20838 | 6036-7156 | 1117 | 0 |
| JXUM01S011970:22157-22993 | 8257-9093 | 837 | 0 |
| JXUM01S011970:18159-18547 | 4227-4619 | 389 | 3.00E-172 |
| JXUM01S047884:1-843 | 443-5285 | 843 | 0 |
| JXUM01S044631:1-851 | 3184-4032 | 851 | 0 |
| JXUM01S012828:16345-17111 | 7695-8460 | 767 | 0 |
| JXUM01S009655:1134-1900 | 7695-8460 | 767 | 0 |
| JXUM01S010445:1-758 | 8236-9000 | 758 | 0 |
| JXUM01S008747:20580-21401 | 5937-6759 | 822 | 0 |
| JXUM01S008747:20246-20578 | 3280-3612 | 333 | 1.00E-158 |
| JXUM01S014308:1-681 | 8165-8846 | 681 | 0 |
| JXUM01S014308:816-912 | 8654-8750 | 97 | 3.00E-40 |
| JXUM01S026498:633-1335 | 3310-4017 | 703 | 0 |
| JXUM01S000231:548499-549126 | 12716-13343 | 628 | 0 |
| JXUM01S007939:1-570 | 8350-8919 | 570 | 0 |
| JXUM01S003856:56829-57416 | 9462-10048 | 588 | 0 |
| JXUM01S002602:932-1531 | 9462-10061 | 600 | 0 |
| JXUM01S005260:83477-84066 | 3364-3951 | 590 | 0 |
| JXUM01S002580:214818-215350 | 5549-6081 | 533 | 0 |
| JXUM01S096607:1-541 | 10920-11460 | 541 | 0 |
| JXUM01S096607:718-912 | 10558-10753 | 195 | 2.00E-87 |
| JXUM01S000757:97513-98075 | 10457-11018 | 563 | 0 |
| JXUM01S007346:21437-21951 | 1515-2029 | 515 | 0 |
| JXUM01S003132:137963-138499 | 10140-10676 | 537 | 0 |
| JXUM01S075904:5060-5545 | 12864-13349 | 486 | 0 |
| JXUM01S002882:162449-162942 | 12520-13006 | 494 | 0 |
| JXUM01S011233:13852-14307 | 9594-10048 | 456 | 0 |
| JXUM01S107719:433-862 | 4420-4849 | 430 | 0 |
| JXUM01S107719:1-204 | 5078-5281 | 204 | 1.00E-76 |
| JXUM01S000489:126264-126723 | 12250-12702 | 460 | 0 |
| JXUM01S013898:10552-10959 | 7695-8102 | 408 | 0 |
| JXUM01S000570:351000-351422 | 13862-14289 | 1423 | 0 |
| JXUM01S001291:169294-169783 | 2868-3382 | 490 | 0 |
| JXUM01S003171:11939-12322 | 13502-13885 | 384 | 0 |
| JXUM01S015849:5036-5409 | 11827-12200 | 374 | 2.00E-175 |
| JXUM01S015849:1-372 | 12280-12644 | 372 | 1.00E-165 |
| JXUM01S025443:1040-1377 | 13025-13362 | 338 | 2.00E-169 |
| JXUM01S117307:1-375 | 4647-5030 | 375 | 7.00E-162 |
| JXUM01S117307:563-838 | 5203-5478 | 276 | 1.00E-107 |
| JXUM01S117307:489-603 | 9484-9596 | 115 | 3.00E-34 |
| JXUM01S000625:141799-142111 | 11517-11828 | 313 | 8.00E-155 |
| JXUM01S003673:102071-102414 | 6083-6425 | 344 | 1.00E-152 |
| JXUM01S003673:101032-101343 | 5774-6085 | 312 | 1.00E-140 |
| JXUM01S136513:1-318 | 2148-2465 | 318 | 4.00E-152 |
| JXUM01S061233:1-307 | 11223-11529 | 307 | 2.00E-143 |
| JXUM01S062495:1-293 | 11390-11681 | 293 | 1.00E-134 |
| JXUM01S063268:1-285 | 7567-7851 | 285 | 3.00E-134 |
| JXUM01S000850:329330-329840 | 1436-1930 | 511 | 3.00E-134 |
| JXUM01S000307:393789-394089 | 15472-15768 | 301 | 3.00E-134 |
| JXUM01S06202:1-298 | 11586-11884 | 298 | 1.00E-133 |
| JXUM01S062160:1-296 | 1349-1642 | 296 | 5.00E-132 |
| JXUM01S003957:77535-77803 | 13280-13547 | 269 | 1.00E-126 |
| JXUM01S000150:406301-406569 | 13280-13547 | 269 | 1.00E-126 |
| JXUM01S031992:495-752 | 8596-8853 | 258 | 6.00E-125 |
| JXUM01S05235:346-600 | 8599-8853 | 255 | 2.00E-124 |
| JXUM01S086674:962-1216 | 8599-8853 | 255 | 2.00E-124 |
| JXUM01S063965:1-277 | 11543-11819 | 277 | 7.00E-124 |
| JXUM01S039912:493-741 | 7719- 7967 | 249 | 9.00E-123 |
| JXUM01S066472:1-252 | 7670-7921 | 252 | 6.00E-119 |
| JXUM01S066164:1-256 | 191-445 | 256 | 2.00E-118 |
| JXUM01S066936:1-249 | 10402-10650 | 249 | 1.00E-114 |
| JXUM01S067339:1-246 | 13157-13407 | 246 | 4.00E-114 |
| JXUM01S067720:1-243 | 12169- 12411 | 243 | 2.00E-111 |
| JXUM01S145863:1-232 | 1752-1983 | 232 | 4.00E-108 |
| JXUM01S002538:134769-134997 | 8201-8429 | 229 | 1.00E-107 |
| JXUM01S002065:1358-1575 | 1-219 | 218 | 9.00E-104 |
| JXUM01S069672:1-229 | 7496-7724 | 229 | 5.00E-101 |
| JXUM01S083965:484-698 | 1255-1470 | 215 | 2.00E-99 |
| JXUM01S151376:1-211 | 9361-9527 | 211 | 3.00E-97 |
| JXUM01S149400:1-218 | 11602- 11817 | 218 | 1.00E-96 |
| JXUM01S068799:1-235 | 3724-3956 | 235 | 1.00E-96 |
| JXUM01S072075:1-214 | 12364-12577 | 214 | 1.00E-95 |
| JXUM01S148981:1-219 | 4422-4640 | 219 | 1.00E-95 |
| JXUM01S067468:1-244 | 5296-5538 | 244 | 6.00E-93 |
| JXUM01S071868:1-215 | 7588-7809 | 215 | 2.00E-92 |
| JXUM01S053160:1-205 | 13192-13396 | 205 | 8.00E-92 |
| JXUM01S14706:1-227 | 10850-11076 | 227 | 3.00E-91 |
| JXUM01S004681:121117-121315 | 7858-8056 | 199 | 3.00E-90 |
| JXUM01S009097:6046-6387 | 4775-5111 | 342 | 4.00E-89 |
| JXUM01S074858:1-201 | 12196-12395 | 201 | 1.00E-88 |
| JXUM01S003186:138053-138276 | 4712-4935 | 224 | 1.00E-88 |
| JXUM01S008551:4167-4352 | 5549-5734 | 186 | 6.00E-87 |
| JXUM01S099115:393-683 | 9912-10200 | 291 | 7.00E-86 |
| JXUM01S099115:1101-1281 | 7695-7875 | 181 | 7.00E-86 |
| JXUM01S000032:401115-401315 | 13965-14168 | 201 | 6.00E-81 |
| JXUM01S001665:267480-267654 | 1492-1666 | 175 | 3.00E-78 |
| JXUM01S003392:51574-51767 | 12134-12335 | 194 | 4.00E-76 |
| JXUM01S03766:291-470 | 5027-5206 | 180 | 1.00E-75 |
| JXUM01S000719:72375-72538 | 8208-8371 | 164 | 5.00E-75 |
| JXUM01S003474:3009-3216 | 1200-1405 | 208 | 2.00E-73 |
| JXUM01S003016:7730-7932 | 4266-4468 | 203 | 2.00E-73 |
| JXUM01S152351:1-207 | 7266-7472 | 207 | 4.00E-70 |
| JXUM01S001778:647-840 | 1200-1396 | 194 | 1.00E-69 |
| JXUM01S001887:135774-135944 | 5229-5399 | 171 | 3.00E-65 |
| JXUM01S000317:388846-388997 | 14247-14398 | 152 | 1.00E-64 |
| JXUM01S068657:92-236 | 8448-8592 | 145 | 7.00E-61 |
| JXUM01S002043:15559-15684 | 1872-1997 | 126 | 2.00E-54 |
| JXUM01S008123:10963-11083 | 4959-5079 | 121 | 1.00E-50 |
| JXUM01S001847:176008-176138 | 2396-2526 | 131 | 1.00E-50 |
| JXUM01S000809:112545-112675 | 2396-2526 | 131 | 1.00E-50 |
| JXUM01S000573:344486-344606 | 4959-5079 | 121 | 1.00E-50 |
| JXUM01S011486:8058-8170 | 11069 -11181 | 113 | 6.00E-49 |
| JXUM01S000239:89378-89495 | 7587-7704 | 118 | 3.00E-46 |
| JXUM01S000105:301137-301269 | 9902-10033 | 133 | 3.00E-46 |
| JXUM01S000924:250294-250413 | 7597-7718 | 120 | 5.00E-44 |
| JXUM01S007401:61509-61621 | 11749-11861 | 113 | 2.00E-43 |
| JXUM01S001003:269286-269393 | 4078-4185 | 108 | 2.00E-43 |
| JXUM01S019872:1691-1792 | 8599-8700 | 102 | 6.00E-43 |
| JXUM01S000175:441557-441678 | 4478-4599 | 122 | 6.00E-43 |
| JXUM01S002412:158482-158587 | 4959-5064 | 106 | 2.00E-42 |
| JXUM01S055370:350-459 | 12209-12318 | 110 | 7.00E-42 |
| JXUM01S015855:3310-3434 | 4333-4459 | 125 | 7.00E-42 |
| JXUM01S008080:13243-13346 | 8326-8429 | 104 | 2.00E-41 |
| JXUM01S006298:76001-76109 | 4615-4723 | 109 | 2.00E-41 |
| JXUM01S014217:9933-10035 | 224-326 | 103 | 8.00E-41 |
| JXUM01S003852:34526-34646 | 12098-12218 | 121 | 3.00E-40 |
| JXUM01S076484:1512-1619 | 8313-8420 | 108 | 3.00E-39 |
| JXUM01S013540:11532-11630 | 4942-5040 | 99 | 1.00E-38 |
| JXUM01S011204:27584-27680 | 13798-13894 | 97 | 1.00E-38 |
| JXUM01S000367:513344-513446 | 2459-2561 | 103 | 4.00E-38 |
| JXUM01S000473:343376-343484 | 1521-1628 | 109 | 1.00E-37 |
| JXUM01S005007:117379-117469 | 13127-13217 | 91 | 5.00E-37 |
| JXUM01S006149:78298-78385 | 2459-2546 | 88 | 2.00E-35 |
| JXUM01S002031:106496-106590 | 12520-12614 | 95 | 8.00E-35 |
| JXUM01S000366:30326-30424 | 7407-7505 | 99 | 3.00E-34 |
| JXUM01S065659:144-261 | 7922-8039 | 124 | 9.00E-34 |
| JXUM01S043906:373-460 | 4887-4974 | 88 | 9.00E-34 |
| JXUM01S000555:259980-260071 | 5852-5943 | 92 | 3.00E-33 |
| JXUM01S010032:25925-26010 | 12142-12227 | 86 | 1.00E-32 |
| JXUM01S009318:3475-3560 | 12142-12227 | 86 | 1.00E-32 |
| JXUM01S002968:188456-188551 | 3675-3770 | 96 | 1.00E-32 |
| JXUM01S002499:20625-20717 | 8734-8826 | 93 | 1.00E-32 |
| JXUM01S002723:131032-131118 | 1435-1521 | 87 | 4.00E-32 |
| JXUM01S000689:212657-212743 | 5985-6071 | 87 | 4.00E-32 |
| JXUM01S003382:22227-22310 | 13191-13274 | 84 | 2.00E-30 |
| JXUM01S001356:192290-192371 | 10579-10660 | 82 | 2.00E-30 |
| JXUM01S008588:49081-49165 | 6011-6095 | 85 | 2.00E-29 |
| JXUM01S003228:127247-127330 | 6066-6148 | 84 | 2.00E-29 |
| JXUM01S002690:131335-131414 | 2450-2529 | 80 | 2.00E-29 |
| JXUM01S002141:191465-191549 | 4337-4421 | 85 | 2.00E-29 |
| JXUM01S000609:234978-235064 | 2893-2979 | 87 | 2.00E-29 |
| JXUM01S006304:48960-49043 | 2255-2338 | 84 | 7.00E-29 |
| JXUM01S003688:151572-151666 | 8392-8486 | 95 | 3.00E-28 |
| JXUM01S000227:280943-281020 | 1456-1533 | 78 | 3.00E-27 |
| JXUM01S001326:171060-171160 | 232-330 | 101 | 1.00E-26 |
| JXUM01S000323:145579-145658 | 12468-12547 | 80 | 1.00E-26 |
| JXUM01S003403:127828-127911 | 10615-10697 | 164 | 5.00E-25 |
| JXUM01S000169:9198-9299 | 1799-1900 | 102 | 5.00E-25 |
| JXUM01S000762:308185-308265 | 2226-2306 | 81 | 2.00E-24 |
| JXUM01S003312:80187-80258 | 12804-12875 | 72 | 6.00E-24 |
| JXUM01S001193:96107-96182 | 13207-13283 | 76 | 6.00E-24 |
| JXUM01S003803:22373-22443 | 11046-11117 | 71 | 7.00E-23 |
| JXUM01S000467:255096-255180 | 4149-4234 | 85 | 2.00E-22 |
| JXUM01S000467:268824-268908 | 4149-4234 | 85 | 2.00E-22 |
| JXUM01S000467:266537-266601 | 4149-4234 | 65 | 2.00E-17 |
| JXUM01S006185:70653-70715 | 1765-1827 | 63 | 8.00E-22 |
| JXUM01S000724:198666-198731 | 4644-4709 | 66 | 8.00E-22 |
| JXUM01S000485:162523-162605 | 215-296 | 83 | 8.00E-22 |
| JXUM01S004410:53895-53976 | 3180-3261 | 81 | 3.00E-21 |
| JXUM01S000141:491366-491427 | 12813-12874 | 62 | 3.00E-21 |
| JXUM01S051484:107-177 | 11046-11117 | 71 | 4.00E-20 |
| JXUM01S026859:857-916 | 2047-2106 | 60 | 4.00E-20 |
| JXUM01S000412:391306-391377 | 3018-3093 | 72 | 4.00E-20 |
| JXUM01S002315:157060-157133 | 11265-11338 | 74 | 1.00E-19 |
| JXUM01S001476:110772-110840 | 3491-3559 | 69 | 1.00E-19 |
| JXUM01S001259:472374-472431 | 12165-12222 | 58 | 4.00E-19 |
| JXUM01S005429:42809-42882 | 3180-3253 | 74 | 5.00E-18 |
| JXUM01S011684:1403-1477 | 33-107 | 75 | 2.00E-17 |
| JXUM01S000979:194917-195000 | 2173-2254 | 84 | 2.00E-17 |
| JXUM01S000085:589938-590009 | 8477-8547 | 72 | 2.00E-17 |
| JXUM01S000009:511513-511589 | 2932-3008 | 77 | 6.00E-17 |
| JXUM01S016112:1845-1907 | 5655-5716 | 63 | 2.00E-16 |
| JXUM01S000378:212405-212478 | 33-107 | 74 | 8.00E-16 |
| JXUM01S002755:68077-68142 | 10816-10881 | 66 | 3.00E-15 |
| JXUM01S000192:103285-103348 | 11396-11459 | 64 | 3.00E-15 |
| JXUM01S001211:179095-179154 | 262-321 | 60 | 9.00E-15 |
| JXUM01S001454:295566-295619 | 1-54 | 54 | 3.00E-14 |
| JXUM01S012733:1142-1186 | 2020-2064 | 45 | 5.00E-12 |
| JXUM01S000578:200415-200455 | 8663-8703 | 41 | 7.00E-10 |
| JXUM01S039344:192-228 | 6102-6138 | 37 | 1.00E-07 |
| JXUM01S000488:82401-82447 | 4732-4778 | 47 | 1.00E-07 |

^a^ Lack of control region.
